# Stellenwert der Radiotherapie bei Morbus Hodgkin in intermediären Stadien: Ergebnisse der HD17-Studie (GHSG)

**DOI:** 10.1007/s00066-021-01826-w

**Published:** 2021-08-11

**Authors:** Christoph Süß, Oliver Kölbl

**Affiliations:** Klinik und Poliklinik für Strahlentherapie, Universitätsklinikum Regensburg, Franz-Josef-Strauß-Allee 11, 93053 Regensburg, Deutschland

## Hintergrund

Bei der zu kommentierenden Arbeit handelt es sich um die Publikation der HD17-Studie der GHSG (German Hodgkin Study Group). Ziel der Untersuchung war es, bei Morbus Hodgkin in intermediären Stadien eine Deeskalation der Therapie durch den Verzicht auf die Radiotherapie (RT) bei PET-negativem Befund nach 4 Zyklen Chemotherapie zu evaluieren. Bei PET-positivem Befund erfolgte eine Involved-node-RT (INRT). Verglichen wurde mit der gegenwärtigen Standardtherapie im intermediären Stadium bei Morbus Hodgkin. Als primärer Endpunkt wurde das progressionsfreie Überleben (PFS) untersucht. Sekundäre Endpunkte waren das Gesamtüberleben (OS), Komplettremission (CR), PET-Negativität nach 4 Zyklen Chemotherapie, Toxizitäten zum Ende der Therapie und das Auftreten von Sekundärmalignomen im Verlauf.

## Patienten und Methoden

In die multizentrische, randomisierte Phase-III-Studie wurden insgesamt 1100 Patienten (Alter: 18–60) aus Deutschland, Österreich, der Schweiz und den Niederlanden mit Morbus Hodgkin im intermediären Stadium eingeschlossen und 1:1 in 2 Gruppen randomisiert. Im PET4-geführten Arm erhielten die Patienten 2 Zyklen eBEACOPP + 2 Zyklen ABVD und im Anschluss, falls das PET daraufhin negativ wurde, keine RT mehr. Nur im Fall, wenn sich nach der Chemotherapie in der PET ein positiver Residualbefund ergab, wurde eine Involved-node-RT durchgeführt. Verglichen wurde mit dem seinerzeitigen Standardarm (2 Zyklen eBEACOPP + 2 Zyklen ABVD, gefolgt von einer Involved-field-Radiotherapie [IFRT] mit 30 Gy). Das Hauptziel war es, eine Nichtunterlegenheit des untersuchten Regimes im Hinblick auf das PFS im PET4-geführten Arm (PET/CT-nach 4 Zyklen Chemotherapie) zu zeigen.

## Ergebnisse

Zwischen 13.01.2012 und 21.03.2017 wurden initial 1100 Patienten*innen eingeschlossen, deren Daten letztendlich in 428 Fällen im Standardarm und 477 im PET4-Arm in der Per-protocol-Analyse ausgewertet werden konnten. Bei einem medianen Follow-up von 46,2 Monaten lag das progressionsfreie Überleben bei 97,3 % (im Standardarm) und 95,1 % (im PET4-geführten Arm). Der Unterschied zwischen den Gruppen lag mit 2,2 % im Rahmen der definierten Grenze von 8 % für Nichtunterlegenheit. Bei 348 (69 %) von 502 Patienten im Standardarm und 290 (57 %) von 507 Patienten im PET4-geführten Arm zeigte sich eine Komplettremission. An Nebenwirkungen (CTC Grad 3 oder 4) traten unter bzw. direkt nach der Chemotherapie vor allem Leukopenien (436/528 Patienten im Standardarm [83 %] vs. 443/529 Patienten im PET4-geführten Arm [84 %]) und Thrombozytopenie (139 [26 %] vs. 176 [33 %]), Infektionen (32 [6 %] vs. 40 [8 %]) sowie Nausea oder Emesis (38 [7 %] vs. 29 [6 %]) auf. Nach Radiotherapie ergaben sich an Akuttoxizitäten vor allem Dysphagie (26 [6 %] im Standardarm vs. 3 [2 %] im PET4-Arm) und Mukositis (9 [2 %] vs. keine). Ein Patient im PET4-geführten Arm verstarb an einer Sepsis.

## Schlussfolgerung der Autoren

Bei Patienten mit neu diagnostiziertem Morbus Hodgkin im intermediären Stadium kann, wenn sich nach der Behandlung mit 2 + 2-Chemotherapien ein PET4-negativer Befund ergibt, auf eine konsolidierende Radiotherapie verzichtet werden, ohne dass damit ein klinisch relevanter Effektivitätsverlust der Therapie einhergeht. Die PET4-geführte Therapie könnte dadurch den Anteil derjenigen Patienten verringern, die nach RT ein Risiko für Spättoxizitäten haben.

## Kommentar

Bei der hier zu kommentierenden Arbeit handelt es sich um die Auswertung der HD17-Studie der GHSG, welche als erste prospektive und randomisierte Studie beweisen sollte, dass eine PET-basierte Indikationsstellung zur konsolidierenden INRT nach Chemotherapie (2 Zyklen eBEACOPP + 2 Zyklen ABVD) bei Patienten mit Morbus Hodgkin im intermediären Stadium keine Einbuße an therapeutischer Effizienz verursacht (Nichtunterlegenheit). Als Vergleich diente die herkömmliche kombinierte 2 + 2-Chemotherapie (2 Zyklen eBEACOPP + 2 Zyklen ABVD) mit anschließender IFRT. Bisherige Arbeiten mit einer ähnlichen Fragestellung hatten mit kleineren Patientenkollektiven diese Nichtunterlegenheit nicht nachweisen können. Sie konnten den primären Endpunkt nicht erreichen (UK RAPID) und/oder behandelten mit anderen Chemotherapieschemata, wobei in die H10-Studie nur frühe und intermediäre Stadien eingeschlossen worden waren. Einzelheiten des H10-Trials der European Organisation for Research and Treatment of Cancer finden sich in der UK RAPID [1, 9].

Die prominente Aussage der Autoren unserer hier kommentierten Analyse ist daher, dass bei PET4-negativem Befund nach einer 2 + 2-Chemotherapie bei Patienten mit Morbus Hodgkin im intermediären Stadium auf eine konsolidierende Radiotherapie verzichtet werden kann. Damit könnte das Risiko von Spättoxizitäten der Radiotherapie zumindest deutlich verringert werden. Es handelt sich hier also um eine weitere wertvolle Erkenntnis aus der international hochgeschätzten GHSG, insbesondere, weil das Patientenkollektiv mit Morbus Hodgkin überwiegend jung war (im Median 31 Jahre).

Als Beleg für die Langzeittoxizität der Strahlentherapie führen die Autoren allerdings Arbeiten des Zeitraums 1964–2004 an, meistens 1965–1995, als die Patienten mit heute veralteten Techniken, z. B. Großfeld-RT, bestrahlt wurden. Deshalb wäre letztendlich noch zu zeigen, ob durch den Verzicht auf die konsoliderende IFRT/INRT mit modernen Techniken tatsächlich Langzeitschäden, insbesondere Sekundärmalignome, verhindert werden können. Denn speziell die angeführten Langzeittoxizitäten wie Hypothyreose, kardiovaskuläre Schäden und Brustkrebs bei jungen Patientinnen mit Morbus Hodgkin können heute mit modernen Techniken (IMRT, VMAT in „Butterfly“-Technik etc.) weitgehend reduziert werden [5, 7].

Hinsichtlich der Toxizität der Chemotherapie geben die Autoren von HD17 zu bedenken, dass eBEACOPP ein intensiveres Behandlungsschema darstellt als ABVD. Denn in der Vorgängerstudie HD14 wurden deutlich mehr Nebenwirkungen bei eBEACOPP als bei ABVD berichtet. So ergaben sich in der Auswertung der HD14-Studie, in der 4 Zyklen ABVD und 2 Zyklen eBEACOPP plus 2 Zyklen ABVD, jeweils gefolgt von einer 30 Gy IF-RT miteinander verglichen wurden, deutliche Unterschiede hinsichtlich der Grad-3/4-Toxizitäten (87,1 % Grad-3/4-Toxizitäten bei eBEACOPP vs. 50,7 % bei ABVD; [12]).

Akute Nebenwirkungen im Sinne von Grad 3 oder 4 traten bei der Durchführung der Chemotherapie bei 455/528 Patienten im Standardarm (86 %) und bei 454/529 (86 %) im PET4-geführten Arm auf. Am häufigsten waren Anämie, Leukopenie, Thrombozytopenie, Nausea bzw. Emesis, aber auch Infektionen unter Chemotherapie. Unter der konsolidierenden Radiotherapie litten nur 37 (9 %) von 429 Patienten im Standardarm unter entsprechenden Akuttoxizitäten CTC Grad 3 oder 4 sowie 4/155 Patienten (3 %) im PET4-geführten Arm, die sich nachträglich noch einer zusätzlichen RT unterzogen hatten.

Darüber hinaus wäre grundsätzlich auch die Auswirkung einer Reduktion der Bestrahlungsdosis auf beispielsweise 20 Gy in Kombination mit der 2 + 2-Chemotherapie im Hinblick auf eine Verringerung der Langzeitnebenwirkungen zu untersuchen. Überhaupt erschien es uns bisweilen unerklärlich, dass bei internistisch ausgerichteten Onkologen oft nur über eine Dosisreduktion bei der Radiotherapie diskutiert wird, nicht aber auch bei den Chemotherapieschemata – insbesondere, wenn man die in der Arbeit genannten Akuttoxizitäten, aber auch Langzeittoxizitäten genauer betrachtet. Immerhin stellt die Radiotherapie seit Beginn der Therapie des Morbus Hodgkin eine wichtige Säule dar. Lediglich für fortgeschrittene Stadien des Morbus Hodgkin wurden kürzlich vielversprechende Ergebnisse hinsichtlich einer Deeskalation der Chemotherapie im Rahmen der HD18-Studie der GHSG publiziert [3]. Die Langzeitdaten werden mit Spannung erwartet.

Im Hinblick auf die Beurteilung von Sekundärtumoren und anderer Langzeittoxizitäten erscheint uns übrigens der mediane Follow-up der hier kommentierten HD17 mit 46,2 Monaten (IQR 32,7–61,2) noch recht kurz. Beschrieben wurden nämlich nur 7 Sekundärmalignome im Standardarm (1 %) und 8 (1 %) im PET4-geführten Arm. Auch wenn diese Sekundärmalignome von den Autoren der Arbeit vornehmlich der Radiotherapie angelastet werden, bleibt natürlich unbeantwortet, wie hoch die Rate an Langzeittoxizitäten bei längerem Follow-up wäre und wie hoch dabei der Anteil der von der Chemotherapie induzierten an der Gesamtzahl.

Vor allem Langzeittoxizitäten spielen insbesondere bei jüngeren Patienten eine große Rolle. Für junge Patienten sind bei Kinderwunsch u. a. die Fertilität bzw. die Gonaden-Funktionalität posttherapeutisch bedeutsam. Bei Frauen zeigte sich in einer Analyse der HD14-Studie hierbei zwar eine eingeschränkte ovarielle Funktionalität, jedoch insbesondere bei Kombination mit GnRH-Analoga kein Unterschied hinsichtlich Fertilität. Bei Männern wurde beim toxischeren 2 + 2-Regime im Vergleich zu ABVD eine längerfristige Beeinträchtigung der Spermatogenese gesehen. Zum Teil kommt es auf lange Sicht wohl zur Erholung der Spermatogenese bei Männern [2, 6, 10]. Auch die Neurotoxizität liegt bei eBEACOPP höher als bei ABVD. Keine Unterschiede ergaben sich im Langzeit-Follow-up hinsichtlich Sekundärmalignomen.

Bezüglich des progressionsfreien Überlebens nach 5 Jahren schneidet der Standardarm mit insgesamt 97,3 % geringfügig besser ab als der PET4-Arm ohne RT (95,1 %). Der Unterschied der beiden Gruppen betrug 2,2 %, eine Nichtunterlegenheit wurde daher angenommen, weil der Grenzwert vorher mit 8 % definiert worden war. Hinsichtlich Tumorkontrolle beschreiben die Autoren einen Progress oder ein Rezidiv während des Follow-up bei 11 (2 %) von 546 Patienten im Standardarm und 19 (3 %) von 550 Patienten im PET4-geführten Arm. Nach Beendigung der Studie zeigte sich bei 348 (69 %) von 502 Patienten im Standardarm und 290 (57 %) von 507 Patienten im PET4-geführten Arm eine metabolische Komplettremission. Der durchaus deutliche Gewinn von 12 % wird von den Autoren nicht diskutiert, könnte aber durchaus auch ein Effekt der zusätzlichen Radiotherapie sein.

## Fazit

Nach den heute vorliegenden Daten ist eine individualisierte PET-geführte Behandlung nach 2 + 2-Chemotherapie mit eventuellem Verzicht auf eine konsolidierende Strahlentherapie für die meisten Patienten mit Morbus Hodgkin im intermediären Stadium gerechtfertigt. Die PET-geführte Therapie reduziert sicher den Anteil von Patienten mit Spättoxizitäten der Strahlentherapie. Die Therapieentscheidung abhängig vom PET nach erfolgter Chemotherapie zu stellen, ist auch in der aktuellen deutschen Leitlinie sowie in der Leitlinie der NCCN verankert [8, 11].

Bei oftmals jungen Patienten ist eine Deeskalierung der Therapie notwendig. Wurde nur mit ABVD behandelt, wird eine konsolidierende Strahlentherapie empfohlen. Gemäß Leitlinien der ESMO sollte die Intensivierung der Therapie (Chemotherapie als auch Radiotherapie) nach 2 Zyklen ABVD PET-basiert erfolgen. So könnte bei PET-negativem Befund auf eine höhere Dosis *(PET-negativ: 1 weiterer Zyklus ABVD* *+* *20* *Gy ISRT vs. PET-positiv: 2 zusätzliche Zyklen eBEACOPP* *+* *30* *Gy ISRT)* und die damit verbundene hohe Toxizität verzichtet werden [4].

Insgesamt scheinen trotz der guten Ergebnisse in der HD17-Studie einige wenige Limitationen des beschriebenen Prozederes angezeigt zu sein:

1. Mögliche Spättoxizitäten der Strahlentherapie konnten wegen des begrenzten Follow-up-Zeitraums nicht evaluiert werden. Daher fehlen entscheidende Informationen, die eine endgültige Bewertung hinsichtlich des Verzichts auf die konsolidierende Strahlentherapie zulassen.
2. Die Techniken in der Radiotherapie haben sich in den letzten Jahren deutlich weiterentwickelt. Daher sind heute ihre Nebenwirkungen schlechter einzuschätzen. Der erhoffte Benefit durch den Verzicht auf eine Radiotherapie bei Patienten mit Morbus Hodgkin im intermediären Stadium könnte schwächer sein als zunächst vermutet.
3. Die Aussagekraft der PET-Positivität auf das progressionsfreie Überleben ist eingeschränkt, da nur Patienten betrachtet wurden, die den kombinierten Standardarm durchlaufen hatten. Eine Kontrolle von Patienten, die keine Strahlentherapie erhalten hatten, fehlte.

Trotz kleinerer Einschränkungen liefert die Analyse der Studienergebnisse von HD17 einen wichtigen Beitrag zur Therapie des Morbus Hodgkin im intermediären Stadium. Insgesamt ist weiterhin die Behandlung in interdisziplinären Zentren im Sinne eines multimodalen Therapiekonzepts mit moderner PET/CT-Bildgebung zur Unterstützung der Therapieentscheidung notwendig.

*Christoph Süß und Oliver Kölbl, Regensburg*
